# Comparison of two normative paediatric gait databases

**DOI:** 10.1186/1476-5918-6-8

**Published:** 2007-07-17

**Authors:** Victoria L Chester, Maureen Tingley, Edmund N Biden

**Affiliations:** 1 Faculty of Kinesiology, Institute of Biomedical Engineering, University of New Brunswick, Fredericton, NB, E3B 5A3, Canada; 2 Department of Mathematics and Statistics, University of New Brunswick, Fredericton, NB, E3B 5A3, Canada; 3 Dept. of Mechanical Engineering, Institute of Biomedical Engineering, University of New Brunswick, Fredericton, NB, E3B 5A3, Canada

## Abstract

The availability of age-matched normative data is an essential component of clinical gait analyses. Comparison of normative gait databases is difficult due to the high-dimensionality and temporal nature of the various gait waveforms. The purpose of this study was to provide a method of comparing the sagittal joint angle data between two normative databases. We compared a modern gait database to the historical San Diego database using statistical classifiers developed by Tingley et al. (2002). Gait data were recorded from 60 children aged 1–13 years. A six-camera Vicon 512 motion analysis system and two force plates were utilized to obtain temporal-spatial, kinematic, and kinetic parameters during walking. Differences between the two normative data sets were explored using the classifier index scores, and the mean and covariance structure of the joint angle data from each lab. Significant differences in sagittal angle data between the two databases were identified and attributed to technological advances and data processing techniques (data smoothing, sampling, and joint angle approximations). This work provides a simple method of database comparison using trainable statistical classifiers.

## Background

One of the main objectives of gait analysis is to identify deviations in a patient's gait from 'normal' movement patterns. A critical component of gait analysis therefore, is the availability of age-matched normative databases. Researchers of paediatric gait typically develop their own normative databases or refer to published data. Substantial data on temporal-spatial [1-6] and kinematic parameters [7-10] are available in the literature for paediatric gait. Existing trunk kinematic [11] and joint kinetic [12-18] databases tend to consist of small sample sizes and are sparser in the literature.

Until recently, we used a database that was developed at the Children's Hospital, San Diego [2]. This large database contains temporal-spatial and joint angle data for 409 gait cycles for children aged 1.0 to 7.0 years old. Trunk and kinetic data were not available in this database. Difficulties in comparing patient data to normative data originating from another lab are partially attributable to differences in marker sets, data processing techniques, and consistency of clinicians. Other disparities arise from advances in computer technology, which have dramatically improved motion analysis systems and data processing capability over the last decade. These differences hamper construction of algorithms to separate 'abnormal' individual gait patterns measured at modern labs from normative gait patterns that were established using older technologies and algorithms. Based on this, we began developing a new paediatric database at our lab using modern instrumentation and numerical algorithms. Of interest, were the differences in normative profiles between the two databases and its affect on gait classification results. Therefore, the objective of this study was to provide a method of comparing the sagittal joint angle data between two normative databases. We compared the new database to the historical San Diego database using statistical classifiers.

## Method

### Participants

Sixty children aged 1–13 years old were recruited from by distributing bulletins campus and local daycare centres. One child was non-compliant during data collection, reducing the sample size to 59 children. These children were divided into two groups: an 'immature' group consisted of 14 children aged less than 3 years, and 2) a 'mature' group contained 45 children aged 3.0 years and older. This age division was based on previous research regarding the onset of mature gait patterns [2]. This assumption was verified using statistical classifiers that could identify mature, normative gait from immature patterns [19]. Ethical approval for this study was obtained from the University Ethics Committee.

### Instrumentation/Apparatus

A Vicon 512 motion capture system (Oxford Metrics Ltd.) with six infrared cameras (JAI 60 Hz interlaced) was employed to track the three-dimensional trajectories of reflective markers placed on the subjects' skin. Markers of 25 mm and 14 mm diameter were used depending on body size to reduce crossover and merging. Each trial was subjectively examined for merges or crossovers of marker trajectories. The calibrated volume was approximately 6.7 m × 2.4 m × 1.5 m. Two force plates (Kistler 9281B21, Kistler Instruments, Winterthur, Switzerland and AMTI BP5918, Advanced Mechanical Technology, Incorporated, Newton, MA, USA) collected the three-dimensional ground reaction forces and moments during each gait cycle. Two digital cameras, a weight scale, and calipers were used to obtain anthropometric measures.

### Procedures

All data collection was conducted at the motion analysis laboratory at the University of New Brunswick (UNB). Twenty reflective markers, representing key anatomical landmarks, were placed directly on the skin of each participant (Table 1). Children were encouraged to perform at least 20 trials if possible. Immediately following completion of the gait trials, the reflective markers were removed and a new segment inertia marker set [20] was applied. Participants were then asked to stand in the anatomical position within a calibration frame, while simultaneous front and side digital photographs were taken. Correct positioning of the body for these images was accomplished through verbal instructions or passive positioning of limbs by parents within the calibration frame. Anthropometric data such as joint width (using calipers), height and mass were also measured [21].

**Table 1:** Marker locations for gait trials

**Marker Location for Dynamic Trials**
Left and right anterior superior iliac spine
Left and right mid-thigh wand
Left and right lateral femoral condyle
Left and right mid-shank wand
Left and right malleolus
Left and right heel
Left and right 2nd metatarsal head
Sacral wand
Left and right shoulder (midway between neck and acromion process)
C7, base of neck

**Additional Markers for Static Trials**

Left and right greater trochanter

### Data Analysis

The biomechanical model consisted of the left and right foot, shank, thigh and the pelvis and trunk segments. Embedded coordinate systems were created using the three non-collinear markers on each segment. The segment-based coordinate systems were transformed to the instantaneous, joint center-based, embedded coordinate systems using alignment data from the static capture trial and joint width measurements. Joint center determination, marker configuration, marker alignment, and kinematics data reduction protocol were identical to Davis et al. (1991) with the exception of the following: 1) the heel marker was used during dynamic trials, and 2) an embedded coordinate system was created at the ankle joint using the long axis of the foot (heel – toe), and the transverse axis of the shank segment. In doing so, the flexion axis of the ankle was aligned with the anatomical frontal plane of the shank.

Cadence, velocity, and percent of cycle spent in single stance were calculated for each successful gait cycle. The single gait cycle, which most closely approximated the individual mean of all gait cycles on these three measures (based on an unweighted, least-squares calculation), was selected as the final trial for analysis [2]. Joint angles were computed using Euler angles in a *yxz *sequence, corresponding to flexion/extension, adduction/abduction, and internal/external rotation. Similar to Sutherland et al. [2], joint angle data were also computed using the projection angle algorithms and then approximated by finite Fourier series using 6 harmonics. Net joint moments and joint power for the hip, knee, and ankle joints were estimated using an inverse dynamics approach. A mathematical model of the human body was used to estimate the segment inertial properties of each child [20]. The required absolute linear and angular velocities and accelerations were calculated from the embedded coordinate systems using a five-point central difference method of derivation [22]. Prior to differentiation, raw coordinate data was filtered using a second order, 6 Hz low-pass Butterworth filter.

### Statistical Analysis: Comparisons with San Diego Database

**Figure 1:**
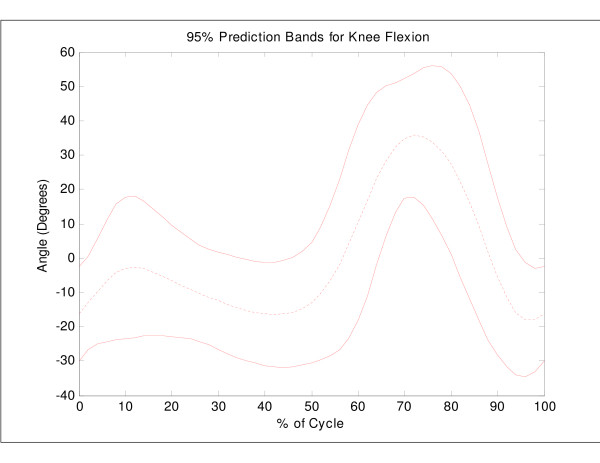
Mean knee flexion angle versus percent cycle for 45 normal subjects, with a 95% bootstrap prediction band. For statistical purposes, the mean of each individual curve was removed prior to computation of the overall mean and bootstrap prediction bands.

**Figure 2:**
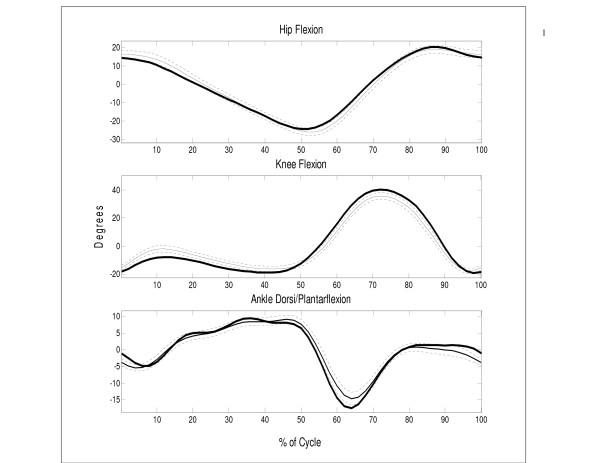
Mean ± (2 S.E.) hip, knee, and ankle joint angles versus percent cycle for UNB normative data (thin lines) with San Diego mean data superimposed (thick lines). For statistical purposes, the mean of each individual curve was removed prior to computation of the overall mean.

**Figure 3:**
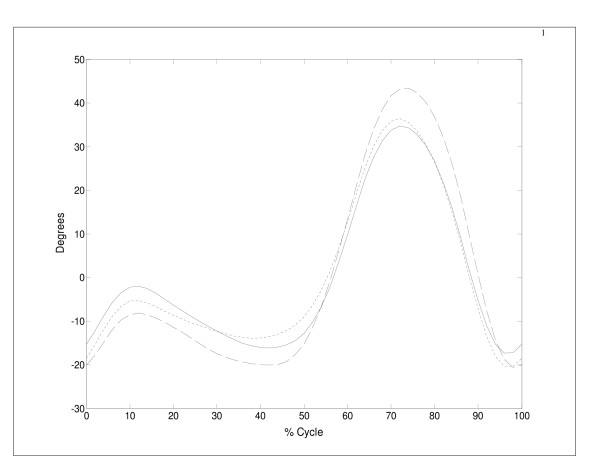
Mean results for knee flexion angles versus percent cycle using UNB's euler (**-**) and projected angle data (---) and San Diego projected angle data (**- -**). For statistical purposes, the mean of each individual curve was removed prior to computation of the overall mean.

**Figure 4:**
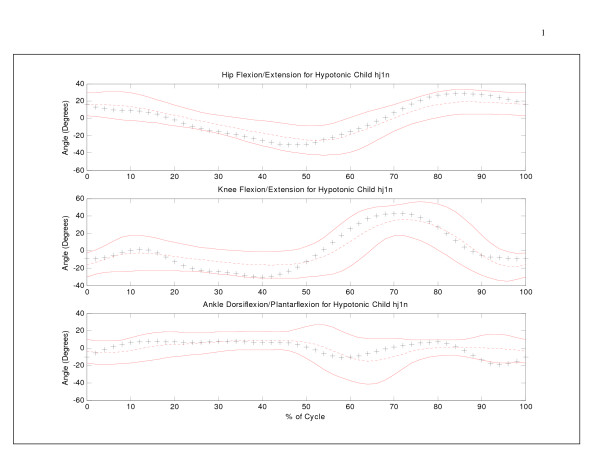
Sagittal hip, knee, and ankle angles (+) versus percent cycle for a hypotonic subject, with 95% bootstrap prediction band. For statistical purposes, the mean of each individual curve was removed for the individual and mean data.

Only the sagittal hip, knee, and ankle kinematics were compared to the San Diego database, for 2 reasons: 1) only kinematic data were readily available for comparison from the San Diego database, and 2) sagittal hip, knee, and ankle joint angles tend to demonstrate greater consistency across labs than do smaller rotations in other planes [23]. The statistical analysis was based on a one-dimensional index of normal gait developed by Tingley et al. [24]. To calculate the index of normal gait, Tingley et al. [24] calculated eleven interpretable functions from the San Diego mature normative data (children aged 3–7 years), namely the mean sagittal joint angle patterns for hip, knee and ankle (3 functions), the mean angular velocities of the three joints (3 functions), the angular acceleration patterns of the three joints (3 functions), and two functions that capture the primary frequencies of knee and ankle angle patterns. To remove bias due to marker misplacement, the classifier subtracts each child's mean angle from the data prior to analysis. This recentred data is used for statistical analyses (Figures 1, 2, 3, 4). A key finding in Tingley's study [24] was that each child's pattern of variation from the group mean could be approximated as a linear combination of these interpretable functions. The gait index developed in this work is simply a squared distance calculated in 11 dimensions (Mahalanobis distance). The gait index classifies children as normal, abnormal, or unusual based on calculations of population percentiles and standard tables of the F distribution [2]. Using this statistical tool, gait patterns of mature children in the UNB normative database were evaluated based on their deviation from San Diego mean normative values.

We expected that the classification of UNB normative data using the San Diego mean normative values would not produce accurate results based on the differences in technology and computational methods between the two databases. Therefore, after classifying the UNB gait patterns using the San Diego mean normative values, the gait index classifier was 'recalibrated' so that each UNB normative gait cycle was classified against the UNB mean normative data (instead of San Diego mean data). New values for the interpretable functions and covariance matrix (required for the distance calculation) were computed. New index scores were calculated for the UNB data and the two sets of classification results were compared. The ability of the recalibrated index to detect abnormal gait patterns was tested by computing the gait index for children under the age of 3 years.

A further examination of the differences between the San Diego and UNB normative data sets was conducted using a multivariate analogue of the two-sample t-test [25]. Differences in the mean and covariance structure of the joint angle data from each lab were investigated. These tests compared differences between 1) San Diego projected angle data and UNB Euler angle data, and 2) San Diego projected angle data and UNB projected angle data. To examine whether the sampling methods and Fourier approximations used by Sutherland et al. [2] were responsible for observed differences in sagittal joint angle data between UNB and San Diego, independent t-tests were used to test for mean differences between raw and smoothed San Diego and UNB data.

The classification results of the index scores for the UNB data were compared to those of the more commonly used bootstrap prediction band methods for assessing clinical cases. Bootstrap techniques (B = 500 samples) were applied to the UNB normative data (n = 83 cycles) to establish prediction regions of normal sagittal knee angle data and ninety-five percent prediction regions (m_*p *_= 3.02) were calculated [26]. A knee flexion curve was considered abnormal if any data point was more than 3.02 standard deviations from the mean curve (Figure 1). The classification results obtained using these bootstrap techniques were then compared to the UNB index scores for a clinical case (hypotonic gait).

## Results

The classification of sagittal joint angle data (Euler method) for children aged 3–13 years at UNB using the classifier based on San Diego mean normative values, resulted in 49% of 83 cycles being classified as unusual or abnormal. When the gait index was recalibrated using the UNB normative data, the new classification results were similar to those of Tingley et al. [24]: the score behaved like an F_11,61 _statistic for the training data, classifying 94% of cycles as normal (as expected by the nature of the training). Further tests using the gait patterns of younger children showed that the recalibrated classifier was also capable of detecting 82% of immature gait patterns (23 out of 28 cycles) at UNB as unusual or abnormal.

The differences between the two gait index results (49% versus 94% classification) were investigated by comparing the mean and covariance structure of the sagittal angles from each database. Both tests yielded highly significant p-values (p = 0.000). Figure 2 shows the UNB mean hip, knee and ankle joint angle curves (± 2 S.E.) with the San Diego mean normative data superimposed. Although the databases appear similar, the two are quite distinct. For example, the peak mean knee flexion between the two databases is more than 2 standard errors apart. When UNB sagittal joint angles were recalculated using a projected angle approach, mean angle patterns were slightly closer to those of San Diego at the beginning of the cycle and midswing (Figure 3), but were still significantly different (p = 0.000).

Data processing techniques between the two labs were suspected to be partially responsible for observed differences between the databases. Sutherland et al. [2] filmed the gait cycles at approximately 50 Hz and later reduced each individual's data to 15–35 evenly spaced frames. The approximation of joint angle curves using Fourier series could yield slightly different results for a curve sampled at 15–35 Hz versus 60 Hz. The results of the independent t-tests of the mean sagittal knee angle (prior to recentering) for both databases showed a significant difference of 8.91° (S.E. ± 1.10°) and 12.04° (S.E. ± 0.89°) for Fourier and raw data, respectively (Table 2). The Fourier approximations actually reduced the difference between the mean knee angles of both datasets.

**Table 2:** Comparison of raw vs smoothed sagittal knee angle across two labs

	** Database**	**N**	**Mean**	**S.D.**	**S.E.**
**RAW DATA**	San Diego	247	70.85	6.46	0.41
	UNB	90	58.81	8.99	0.95
**FOURIER**	San Diego	243	68.40	6.24	0.40
	UNB	90	59.49	9.67	1.02

A comparison of the index scores and the bootstrap prediction bands revealed differences in classification results for clinical gait data. Sagittal hip, knee, and ankle angle data for a hypotonic child [19] are shown in Figure 4 with respect to the 95% prediction range. Both knee and ankle data were classified as 'unusual' and 'abnormal' by the UNB index score, but not by the 95% prediction bands. The main reason for the discrepancy in classification between the two methods is that the predictions bands simply analyzed deviations in magnitude and do not consider differences in the pattern of motion. Unless data points deviate from the mean curve by more than 3.02 standard deviations, the child will not be detected. Only a few ankle angle data points extend beyond the boundaries during terminal swing in Figure 4. However, the three graphs show temporal delays in angle data generating a different pattern of motion compared to normative data. The UNB index scores detected this difference in the shape of the curve.

## Discussion

Differences in normative gait databases are difficult to assess due to the high-dimensionality and temporal nature of the various kinematic waveforms. The purpose of this study was to provide a method of comparing the sagittal joint angle data between two normative databases. We compared a modern gait database to the historical San Diego database using statistical classifiers developed by Tingley et al. [24]. Differences between the two normative data sets were explored using the index scores, and the mean and covariance structure of the joint angle data from each lab [24]. In addition, the boundaries of normality established by the statistical classifier were compared to Bootstrap methods.

The significant differences found between the San Diego and UNB normative databases are likely due to multiple factors. Since the databases were established over 20 years apart, technological differences are most likely a predominant factor. Sutherland et al. [2] used a pseudo three-dimensional system that consisted of four independent 16 mm motion picture cameras, a Vanguard motion analyzer and a Graf-pen sonic digitizer, requiring the manual digitization of images and joint centers. In contrast, UNB used a three-dimensional system with 6 high-resolution cameras, semi-automatic marker digitization and labeling, and automated joint center estimation techniques.

Changes in data processing techniques over the last two decades are also likely a major cause of differences between the two normative datasets. The results of the multivariate analyses showed that the data sets differ in part due to the algorithms used to calculate the joint angles. UNB's normative data showed more similarity to the San Diego values when joint angles were calculated as projected angles, similar to Sutherland et al. [2], as opposed to Euler angles. In addition, comparisons of sampling rates and smoothing techniques showed that the Fourier approximations were related to decreases in the mean amplitude of the knee angle data. San Diego mean sagittal knee angle data was approximately 9° higher than UNB mean knee angle data when Fourier approximations were compared. In contrast, raw angle comparisons generated a difference of approximately 12°.

Once normative data has been collected, establishing bounds of normality for each gait parameter is necessary. Bootstrap methods may be used to establish prediction bands of normality [26,27]. This technique captures large point-wise deviations from the mean of the training data set. It does not necessarily capture deviations in patterns of motion, nor does it consider correlations between curves (i.e. knee and ankle angle). The UNB index scores are capable of classifying gait data based on magnitude of deviation, pattern of motion, and correlations between multiple joint angle curves. Therefore, the UNB classifier is able to extract more complex features of each gait cycle that may be missed by bootstrap methods. The finding that the calibrated index of normality identified 80% of cycles for children under 3 years old as unusual or abnormal supports the findings of Tingley et al. [24] that the eleven interpretable functions can successfully classify gait patterns.

Given that only robust sagittal angles were compared in this analysis, it is likely that the differences between labs would be greater for the other planes of motion. The clinical significance of the differences found between the UNB and San Diego databases in this study are uncertain. However, in light of these differences, we will continue efforts to expand the new normative database and retrain the gait classifier as individuals are added.
